# Natural variation in stress response gene activity in the allopolyploid *Arabidopsis suecica*

**DOI:** 10.1186/s12864-017-4067-x

**Published:** 2017-08-23

**Authors:** Keisha D. Carlson, Noe Fernandez-Pozo, Aureliano Bombarely, Rahul Pisupati, Lukas A. Mueller, Andreas Madlung

**Affiliations:** 1 Department of Biology, University of Puget Sound, 1500 N Warner St, CMB 1088, Tacoma, WA 98416 USA; 2 Boyce Thompson Institute, 533 Tower Road, Ithaca, NY 14853 USA; 3 Gregor Mendel Institute of Molecular Plant Biology, Dr. Bohr-Gasse 3, 1030 Vienna, Austria; 4 Present Address: Virginia Tech, Department of Horticulture, 216 Latham Hall, Blacksburg, VA 24061 USA

**Keywords:** Allopolyploidy, *A. suecica*, Divergence, Stress, Polyploid

## Abstract

**Background:**

Allopolyploids contain genomes composed of more than two complete sets of chromosomes that originate from at least two species. Allopolyploidy has been suggested as an important evolutionary mechanism that can lead to instant speciation. *Arabidopsis suecica* is a relatively recent allopolyploid species, suggesting that its natural accessions might be genetically very similar to each other. Nonetheless, subtle phenotypic differences have been described between different geographic accessions of *A. suecica* grown in a common garden*.*

**Results:**

To determine the degree of genomic similarity between different populations of *A. suecica,* we obtained transcriptomic sequence, quantified SNP variation within the gene space, and analyzed gene expression levels genome-wide from leaf material grown in controlled lab conditions. Despite their origin from the same progenitor species, the two accessions of *A. suecica* used in our study show genomic and transcriptomic variation. We report significant gene expression differences between the accessions, mostly in genes with stress-related functions. Among the differentially expressed genes, there are a surprising number of homoeologs coordinately regulated between sister accessions.

**Conclusions:**

Many of these homoeologous genes and other differentially expressed genes affect transpiration and stomatal regulation, suggesting that they might be involved in the establishment of the phenotypic differences between the two accessions.

**Electronic supplementary material:**

The online version of this article (doi:10.1186/s12864-017-4067-x) contains supplementary material, which is available to authorized users.

## Background

Allopolyploids are a special type of hybrid whose genomes consist of subgenomes from two or more species [1]. Allopolyploids are frequent in nature, especially among cultivated plants [2]. Allopolyploidization has been suggested as an important mechanism for evolution [3–5]. Established allopolyploids are often more vigorous than their progenitor species and, like in diploid hybrids, this phenomenon has sometimes been described as hybrid vigor. However, early generation allopolyploids, like some diploid hybrids, frequently display phenotypic instabilities and low fitness [6]. Both hybrid vigor and hybrid instability have been attributed to genomic upheaval or “genomic stress” as a consequence of the merger of genomes [7]. In the case of allopolyploids such incompatibilities are resolved over time [8]. Eventually allopolyploids may benefit from various genomic and epigenetic changes sustained as a result of allopolyploidization. How and when the resolution of conflict between two genomes in a neoallopolyploid is achieved is still largely unknown.

Because formally allopolyploids have paired homologous chromosomes and in principle are able to undergo normal meiosis, allopolyploidy has been described as a mechanism for “instant speciation” [9]. Accepting this tenant, the first generation of the neoallopolyploid would therefore constitute a new, stable and uniform species, yet genome duplication coupled with genome merger often coincides with non-additive changes that create a newly combined genome with different properties than would be expected from the sum of its parental contributions. Reported changes include alterations in the allopolyploid with respect to gene expression [10–14] and the epigenetic landscape [15–18], as well as genome rearrangements [19–23] making the allopolyploid not only different from the parent species but potentially creating an effective mechanism for reproductive isolation from the parent species.

It is still unclear with what frequency neoallopolyploids re-establish a stable genome and thus become the founders of a stable new species [24, 25]. Experiments with synthetic neoallopolyploids in *Brassica* and *Arabidopsis* have suggested that from an interspecies polyploid cross only few individuals, if any, stabilize their genomes throughout the subsequent generations, while the majority of neopolyploids is either inviable or infertile [26–29]. If genomic stability is not restored soon after allopolyploidization, and instability, for example during meiosis, is still present in subsequent generations then genomic errors, such as aneuploidy, can compound from generation to generation. This spiraling process has been referred to as the “polyploid ratchet”, where genomic errors accumulate over several generations until fitness is compromised to the point of extinction of the line [26]. If extinction does not occur, any genetic or epigenetic changes that accumulate differentially between accessions may of course also have an effect on gene expression.

We used two natural accessions of *A. suecica* to address the question if in this system allopolyploidization has led to transcriptomic and genomic variation between populations. *A. suecica,* native to Scandinavia, is a natural allopolyploid derived from the hybridization of *A. thaliana* and *A. arenosa,* which most likely occurred an estimated 16,000 years ago [30]. Phenotypic analyses of accessions collected throughout Sweden and Finland showed that populations differ from each other in several morphometric traits, flowering time, the occurrence of certain floral abnormalities, and the genomic location of the DNA transposon *Sunfish* [31, 32]. It is, however, not known to date how much variation on the transcriptome level has accumulated between populations in this relatively recent allopolyploid.

Here, we used two populations of *A. suecica* to assess their differences in gene activity under optimal growing conditions. We found a number of differentially expressed transcripts between accessions, especially among stress-related genes. We further report a surprisingly large number of differentially expressed homoeologous gene pairs whose transcription seems to be synchronized within each accession, consistent with the hypothesis that they might play a role in providing selective advantages for each accession in their respective environments.

## Methods

### Plant material, RNA extraction and sequencing

Two previously described accessions of *A. suecica,* Sue 1 and Sue 16 (ABRC accession numbers CS22505, CS22516, respectively) were used in this experiment. Per pot, 4–5 seedlings were grown in soil (Sungrow, Horticulture, Vancouver, Canada) in 20 separate 10 cm diameter pots placed in two plastic trays side by side. The plants were kept on a plant growth rack indoors at relatively constant room temperature (~ 20 °C) with supplemental fluorescent lighting (100 μM photons/m^2^ s^−1^) in a 16 h light/8 h dark photoperiod. Pots were randomized within the two trays during growth. At the time of harvest, tissue pools were formed by excising a single leaf from 3 to 4 plants of a single accession grown in at least two different pots, flash frozen in aluminum foil packets, and stored at −80 °C until use. RNA was extracted from these tissue pools where each pool later formed one biological replicate. A total of three biological replicates of each accession (six samples total) were sent to the Michigan State University Genomics Facility for library construction and RNA-seq sequencing. Illumina libraries were constructed using a TruSeq Standard mRNA library kit with an average insert size of 515 bp, pooled, and run in a single Illumina HiSeq 2500 Rapid Run flow cell. Paired end reads of 2 × 150 bp length (150 PE) were obtained, bases were called using Illumina Real Time Analysis software (version 1.17.21.3), and reads were de-multiplexed using Illumina Bcl2Fastq software (version 1.8.4). Due to lower than expected yield during the first run, the same libraries were run on a second flow cell lane using the same protocol. Reads from both runs for each sample were combined in concatenated files resulting in a total of 27 to 41 million reads (average ~ 34 M reads) per sample. Sequence summary statistics can be found in Additional file 1: Table S1.

### SNP analysis

To get an idea of the amount of genetic variation within the transcriptome of the two tested populations, we determined the amount of SNP differences between the two accessions. We used a combination of read mapping with Bowtie to a synthetic *A. suecica* -like reference sequence, a custom Perl script to separate reads from the homoeologous genes into separate bins, the SNP calling tool from the package Freebayes, and another custom script to display and analyze the Freebayes output files. We started by downloading the freely available genomic DNA contigs from the minimally assembled *A. arenosa* genome. These sequence data were produced in 2011 by the Functional Genomics of Plant Polyploidy group (http://comailab.genomecenter.ucdavis.edu/index.php/The_A._arenosa_genome).

We concatenated this dataset with a genomic *A. thaliana* DNA file to create the synthetic reference genome. The reads were mapped to the indexed concatenated genomic reference using Bowtie2. The resulting .bam files were sorted with samtools. Using the custom Perl script SeparateHomeolog2Sam (https://github.com/aubombarely/GenoToolBox/blob/master/SeqTools/SeparateHomeolog2Sam) we separated the mapped homoeologous reads of one of the biological replicates of RNAseq data of each accession into separate files [33], effectively removing the *A. arenosa* contribution to the transcriptome. This process left us with only the *A. thaliana*-derived, mapped transcripts from *A. suecica.* Using Freebayes software we called SNPs and small indels between the two *A. suecica* accessions and the reference.

To ascertain that *A. arenosa* reads would map correctly to the *A. arenosa* derived part of the genome, we added an RNAseq data file of 50 bp single Illumina reads of leaf tissue from one 4-week-old autotetraploid *A. arenosa* individual (kindly supplied pre-publication by Drs. Ben Hunter and Kirsten Bomblies) to our analysis. Seeing that they did map, these reads were not used in the subsequent analysis. SNP files for each sample were merged and displayed for comparison between samples using the custom Perl script MultiVcfTool (https://github.com/aubombarely/GenoToolBox/blob/master/SeqTools/MultiVcfTool) [33]. Indels were removed and SNPs were counted for pairwise comparisons between samples. The SNPs derived from the *A. thaliana* genome complement were compared to the SNP data set of the 1001 genome project (1001genomes.org). Finally, we used the software tool SNPMatch (https://github.com/Gregor-Mendel-Institute/snpmatch) (Pisupati et al., submitted) to determine which of the accessions from among the 1001 genome project are the closest to the *A. thaliana* genome complement and thus might have had a common ancestor with the *A. thaliana* ecotype that contributed its genome to *A. suecica*. For SNPMatch analysis, the SNPs derived from the *A. thaliana* genome complement in *A. suecica* were further filtered for quality (quality score > 30).

To determine if SNPs were enriched in differentially expressed genes (DEGs) between Sue 1 and Sue 16, a chi-square test was performed using R (version 3.1.3). The total number of base pairs in the *A. thaliana* transcriptome was determined using the cDNA fasta file on TAIR. The total number of basepairs in the DEGs was then determined as well as the number of SNPs that fell in those DEGs. This distribution was compared against the null hypothesis that the SNPs were distributed randomly in the transcriptome.

### RNA-seq analysis

Sequence reads were inspected for quality using FastQC and adapters trimmed off using fastq-mcf [34]. A reference genome was created by concatenating cDNA files from *A. thaliana* (TAIR10 version) and *A. lyrata*, a congener of *A. arenosa* with a high quality genome assembly (Phytozome.org, v.1.0). Reads from all 3 biological replicates were mapped against an index file of the synthetic reference using Bowtie2 and Tophat2 software with default parameters [35]. DEGs were identified and analyzed using Cuffdiff with the default settings for the determination of statistical significance [35]. Sequence summary statistics are reported in Additional file 1: Table S1.

### Gene ontology enrichment analysis

DEGs were assigned unique gene IDs wherever possible (TAIR). Gene ontology (GO) enrichment analysis was performed using topGO in R [36]. *P*-values are from Fisher’s Exact Tests using the weighted model [37]. These *p*-values are uncorrected because the parent-child relationships of GO terms are taken into consideration with the weighted model resulting in tests that are not truly independent [36]. We present all GO terms with enrichment *p*-values < .05 and at least two genes in the DEG set annotated with the term.

We further grouped the significant GO terms into four categories: not stress related, abiotic stress response related, biotic stress response related, and general stress response related. The latter category includes GO terms related to both abiotic and biotic stress responses. We then placed the genes that were annotated with the significant GO terms into the four categories. If a gene was annotated with a combination of biotic, abiotic, and general stress response GO terms, the gene was categorized as a general stress response gene.

### Homoeologous gene enrichment analysis

We found a large number of DEGs that occurred in homoeologous pairs. To determine if differentially expressed homoeologous gene pairs compared to any DEGs not occurring in pairs were overrepresented in the complete set of DEGs, we performed permutation tests using R. From complete gene lists of *A. thaliana* and *A. lyrata* obtained from Cuffdiff output (including genes with *A. thaliana* gene IDs and without), a random set of genes equivalent in number to the DEGs from our experiment was drawn and the number of homoeologous pairs in these sets was determined. This was repeated 1,000,000 times and the greatest number of homoeologous gene pairs recovered in the permutation test was recorded.

### Subgenome expression analysis

*A. thaliana* gene IDs were assigned to 13,394 homoeologs between *A. thaliana* and *A. arenosa*. For those 13,394 genes, ratios of average FPKMs (Fragments Per Kilobase of transcript per Million mapped reads) (*A. thaliana* FPKM / *A. lyrata* FPKM) across all biological replicates were calculated in Sue 1 and Sue 16 and plotted using R. Genes with FPKM of zero in one or both subgenomes were excluded (707 genes in Sue 1, 735 genes in Sue 16).

## Results

### Gene expression varies between the accessions

Geographically isolated accessions of *A. suecica* have slightly divergent phenotypes [31, 32]. The two chosen accessions for our comparison, Sue 1 and Sue 16, differ in growth habit, plant size, and flowering time [32]. Our RNAseq analysis found 148 genes to be statistically significantly differentially expressed between Sue 1 and Sue 16 (Additional file 1: Table S2). Of these genes 61% (90) were upregulated in Sue 16, and 39% (58) were upregulated in Sue 1. Gene ontology analysis sorted these genes into 34 statistically significantly enriched categories (Additional file 1: Table S3). Interestingly, many of these categories contain stress-related genes in the broadest sense, including genes responsive to chitin, wounding, or mechanical stimulus, as well as genes involved in biosynthesis or signal transduction of the classical stress response hormones ethylene, abscisic acid (ABA), and jasmonic acid (JA) (Additional file 1: Table S3).

**Fig. 1:**
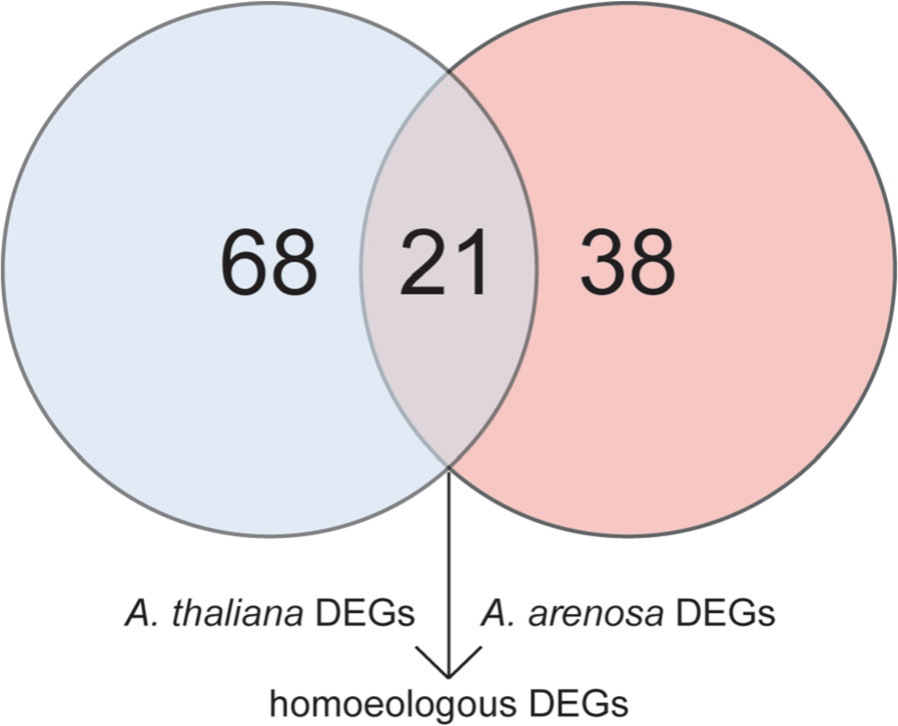
Sister lines derived from the same allopolyploidization event display differential gene expression. Among the 148 (68 + 38+ 2*21) differentially expressed genes (DEGs), 89 (68 + 21) came from the *A. thaliana* subgenome, 59 (21 + 38) came from the *A. arenosa* subgenome. Of these DEGs, 21 gene were differentially regulated in both subgenomes (homoeologous pairs). Interestingly, in all 21 cases both genes in homoeologous pairs were differentially regulated in the same direction (i.e. up or down)

In order to assess from which subgenome differentially expressed homoeologs were being expressed, we needed to map reads back specifically to each parent genome. While for *A. arenosa* only a smaller, pre-publication genome assembly exists, its closely related congener [38] *A. lyrata* is fully sequenced, assembled, and relatively well annotated [39]. *A. lyrata’*s genome has therefore frequently been used in the past as a stand-in for *A. arenosa* [30, 40, 41]. *A. lyrata* and *A. thaliana* however diverged some 10 Mio years and share only about ~83% sequence identity among their RNA [39]. Using the *A. lyrata* genome as a proxy for the paternal *A. arenosa* genome, we mapped the RNA reads to a synthetic reference genome consisting of the concatenated genomes of *A. thaliana* and *A. lyrata.* Of the 59 differentially expressed genes mapped to *A. lyrata* (and thus assumed to be the *A. arenosa* homoeologs), 42 could be assigned gene IDs based on a homology search to the *A. thaliana* genome database (The Arabidopsis Information Resource; TAIR), while 17 remained unassigned. We mapped 89 of the differentially expressed genes between Sue 1 and Sue 16 to the *A. thaliana* genome (7 of them could not be assigned a TAIR gene ID). Of all 124 differentially expressed genes with an assignable ID from the TAIR database, 42 belonged to homoeologous pairs that were differentially expressed between the two accessions in both subgenomes of the *A. suecica* genome (Fig. 1). Permutation analysis showed that finding a number of homoeologous gene pairs this high from among only 148 DEGs by random chance is highly unlikely (*p* < 10^−6^). A trivial explanation could be that sequence reads were ambiguous between the two subgenomes and therefore mapped equally between the two homoeologs. However, both homoeologs were independently found to be significantly differentially expressed, so if all of the ambiguous reads in truth came from one homoeolog, then that homoeolog would have to be extremely differentially expressed between Sue 1 and Sue 16.

**Fig. 2:**
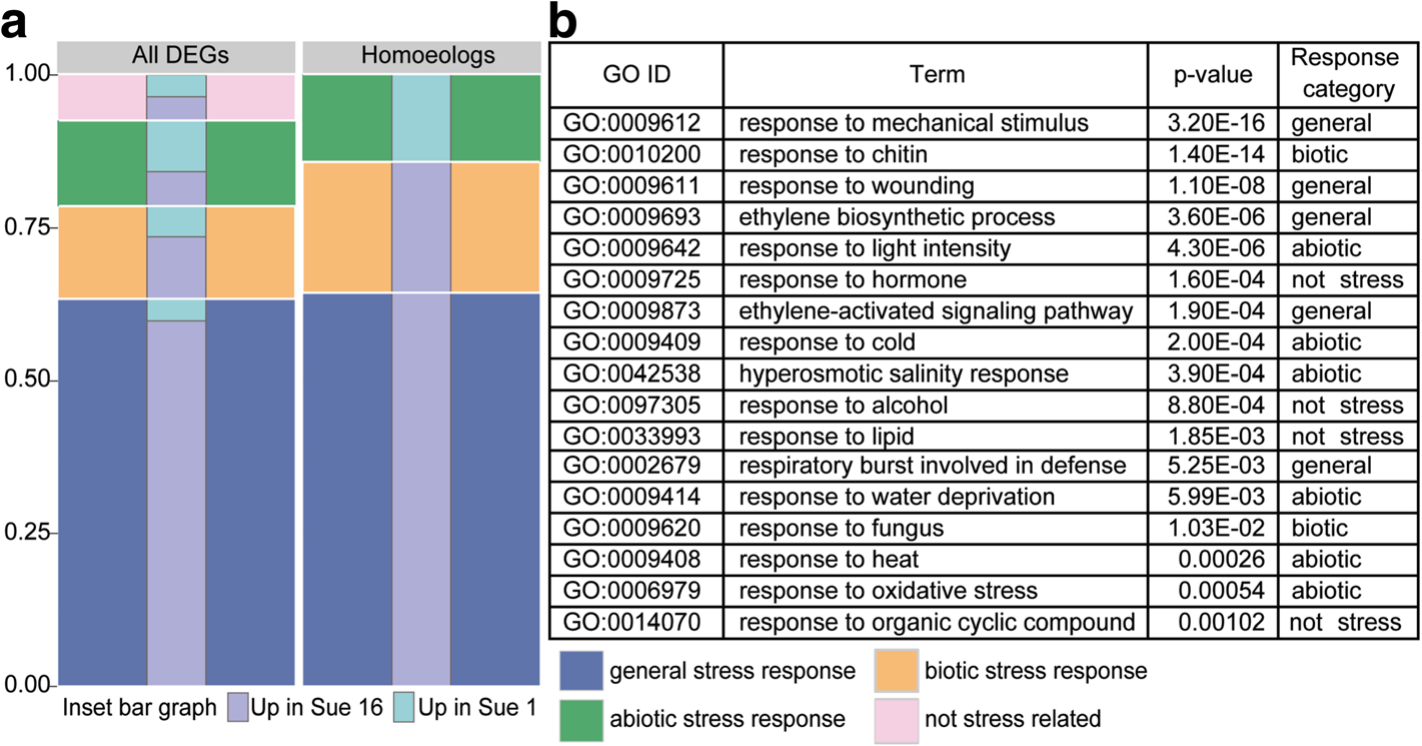
Allopolyploid sister lines differ in their stress responses. **a** Out of the 148 DEGs 79 were found to be enriched in specific Gene Ontology (GO) categories (left panel). Out of the 21 homoeologous DEG 14 genes were significantly enriched in GO categories (right panel). The inset bar displays the proportion of those genes upregulated in Sue 1 (teal) and Sue 16 (purple). **b** Table showing the enriched GO categories for the 21 homoeologous gene pairs and the stress response category assigned to each

Of these 42 genes, 76% (32) were upregulated in Sue 16, while only 24% (10) were upregulated in Sue 1. Interestingly, all of the 21 homoeologous pairs were differentially regulated in the same direction in *A. suecica*, such that both the A. sue - AT homoeolog and the A. sue - AA homoeolog differing in expression between Sue 1 and Sue 16 were regulated the same way (either both up or both down). It is therefore appealing to speculate that these 42 synchronously regulated genes might be under some special selective pressure. If these gene expression differences present a selective advantage for one of the two accessions, then this might present a possible explanation for the observation that both homoeologs in these cases are regulated the same. For these genes, it is also likely that the synchronous changes in gene expression of homoeologs between accessions is due to *trans*-acting mutations (such as transcription factors) regulating both homoeologs, as opposed to *cis*-acting mutations, which would have to accumulate in both homoeologous copies separately to explain the synchronous regulation. As for the entire data set of differentially expressed genes, GO analysis of these 21 gene pairs also found statistically significant enrichment of stress-related GO terms (Fig. 2).

To further compare GO term enrichment in all 148 DEGs to that in the 21 homoeologous pairs, GO terms were identified as general stress related, abiotic stress related, biotic stress related, and not stress related (Fig. 2, Additional file 1: Table S3). The genes that led to enrichment of these GO terms were then similarly categorized. The homoeologous gene set does not contain any significant genes that are not stress related, further highlighting the potential functional relevance of these genes in adaptive differences between the two lines (Fig. 2). Also of note is that biotic and general stress response genes are more often upregulated in Sue 16 relative to Sue 1 whereas abiotic stress response genes are more often upregulated in Sue 1. This could indicate adaptation to different stresses by the two sister lines.

**Fig. 3:**
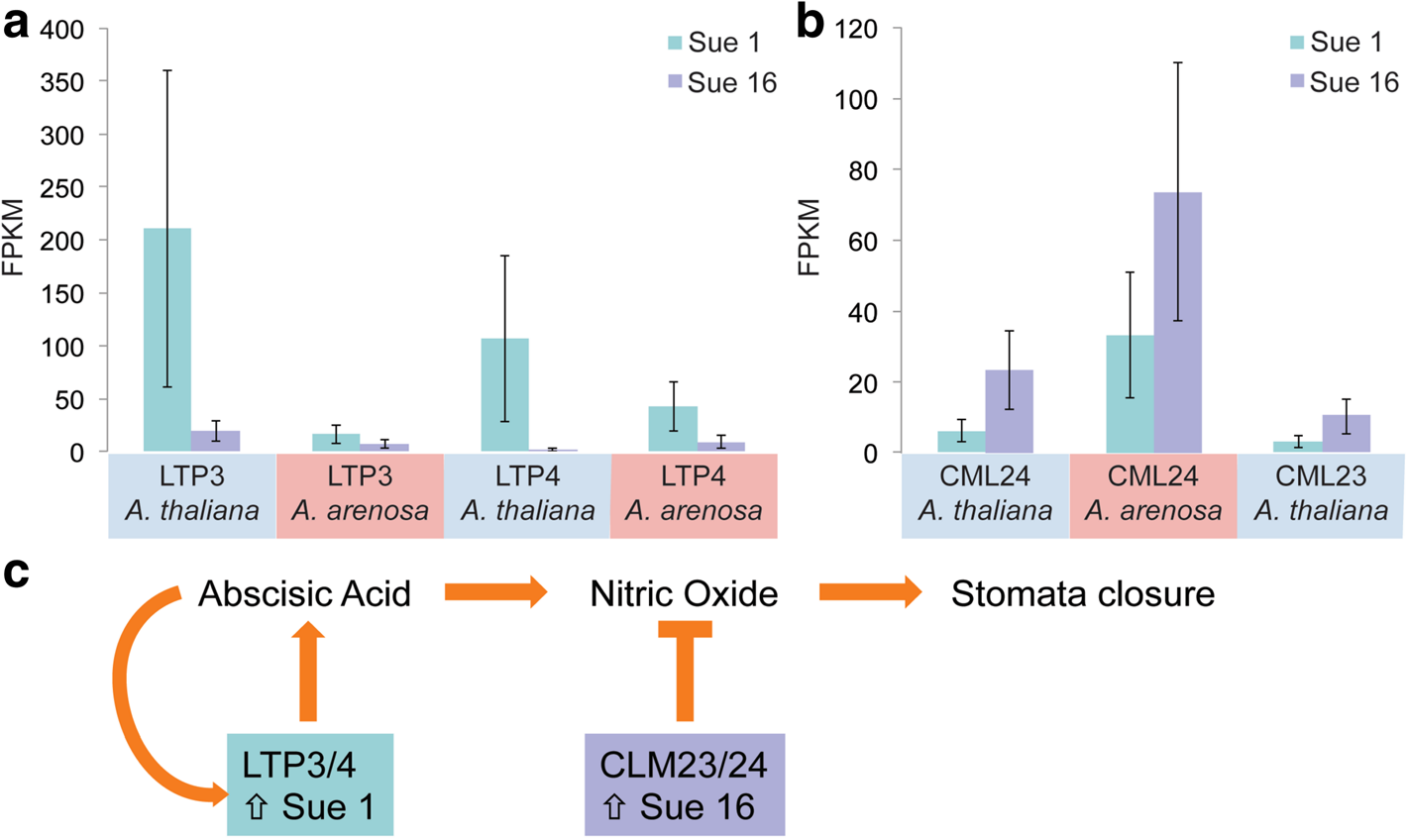
The lipid transfer protein (LTP) family and calmodulin-like (CML) family are significantly and synchronously differentially regulated in Sue 1 and Sue 16. Shown are FPKM expression values with confidence intervals from Cuffdiff analysis for **a** both homoeologs of *LTP3* and *LTP4* and **b** both homoeologs of *CML24,* and the AT homoeolog of *CML23*. **c** A model showing the potential effects of upregulation of *LTP*s in Sue 1 and upregulation of *CML*s in Sue 16 on stomata closure

Any of the 148 differentially expressed genes could potentially have an effect on phenotypic variation between the two accessions. Since differential regulation in the 21 synchronously regulated homoeologous pairs (Additional file 1: Table S2) suggests special importance for these genes, we took a closer look at the functions of some of them. We noticed that among the homoeologs, there were multiple members of a few gene families. For example, both homoeologs of two lipid transfer proteins (*LTP3* and *LTP4*), involved in ABA signaling, and abiotic stress responses [42], were among the significantly differentially expressed genes, and both were upregulated in Sue 1 compared to Sue 16 (Fig. 3a). ABA is a major plant hormone involved in mediating responses to drought and other stresses. ABA transport into the plant’s guard cells leads to stomatal closure [43], which reduces both water loss and gas exchange. *LTP3* has been reported to be expressed in response to ABA and exposure to drought, and *LTP3* is also involved in cuticular wax biosynthesis [44]. Both *LTP3* and *LTP4* appear to act redundantly in ABA biosynthesis [42], maybe via a feedback loop. Overexpression of *LTP3* is correlated with increased ABA levels [42]. Sue 1 showed higher expression levels of both homoeologs of *LTP3* and *LTP4* compared to Sue 16, suggesting that the perceived or chronic stress level in Sue 1 was higher than in Sue 16 leading to increased ABA synthesis.

Another homoeologous pair that was differentially expressed between the two accessions was *CALMODULIN-LIKE 24* (*CML24*). Additionally one of the homoeologs of *CML23* was also upregulated in Sue 16 over Sue 1 (Fig. 3b). *CML23* and *CML24* are involved in flower induction, acting apparently both in the photoperiodic pathway via *CONSTANS* and in the vernalization pathway via *FLOWERING LOCUS* (*FLC*) [45]. Plants mutant for *CML24* flower late, and have increased levels of nitric oxide and the floral repressor *FLC,* while gain-of-function *clm24* mutants flowered early [45]. Given that Sue 16 flowers about 2 weeks earlier than Sue 1 [32], and that expression of *CML23* and *CML24* in Sue 1 is significantly lower than in Sue 16, it is possible that this transcriptional change plays a role in the emerging phenotypic variation between the two accessions. Interestingly, *cml23/cml24* double mutants have also been reported to have elevated levels of nitric oxide (NO) [45], a compound which has both been implicated in oxidative stress defense and acting in concert with ABA in the promotion of stomatal closure [43] (Fig. 3c **)**.

Two additional pairs of homoeologs that are highly upregulated in Sue 16 compared to Sue 1 are the xyloglucan transglycosylases/hydrolases *XTH4* and *XTH22*, which are involved in cell wall modification required for cell elongation and growth [46] (Additional file 2: Figure S1).

Next, we tested if overall gene expression was similar in the two subgenomes of the two accessions. We plotted the relative expression ratio of all *A. thaliana* and *A. arenosa* homoeologs (A. sue AT and A. sue AA, respectively) in both accessions (Additional file 3: Figure S2), and found that overall expression ratios were very similar to each other in Sue 1 versus Sue 16, suggesting the two sister lines utilize the subgenomes similarly. We also noticed that overall relative expression of the A. sue - AT subgenome was unexpectedly almost two-fold greater than that of the A. sue - AA subgenome (Additional file 3: Figure S2). It is possible that some A. sue - AA reads did not map to the synthetic reference genome due to differences between the *A. arenosa* reads and the *A. lyrata* reference. This would indeed have resulted in lower overall FPKM values for *A. arenosa*. However, to explain the 2-fold difference in FPKM value we observed (Additional file 3: Figure S2), around 50% of *A. arenosa* reads would have had to be unmappable. We think this is unlikely, given the close relationship between *A. arenosa* and *A. lyrata* [38]. Our data therefore suggest that the A. sue - AT subgenome overall is more actively transcribed in the two tested *A. suecica* accessions.

Lastly, to address the potential effect of differential SNP accumulation between the two accessions on gene expression, we asked if differential expression of genes between accessions was driven by the accumulation of SNPs within the significantly differentially expressed genes. To test this hypothesis, we calculated the *p*-value from a Chi-squared test, which asked if SNPs were statistically significantly enriched in differentially expressed genes compared to all other genes in the genome (Additional file 1: Table S4) but found no enrichment (*p* = 0.32), suggesting that coding SNPs do not explain the gene expression differences observed between Sue 1 and Sue 16.

Taken together, our data show that the two accessions differed in the expression of only a modest number of genes (Additional file 4) but that these genes and their apparently concerted regulation in multiple homoeologous gene pairs within each line may be of functional importance.

### Geographical accessions of *A. suecica* also display genomic variation

**Fig. 4:**
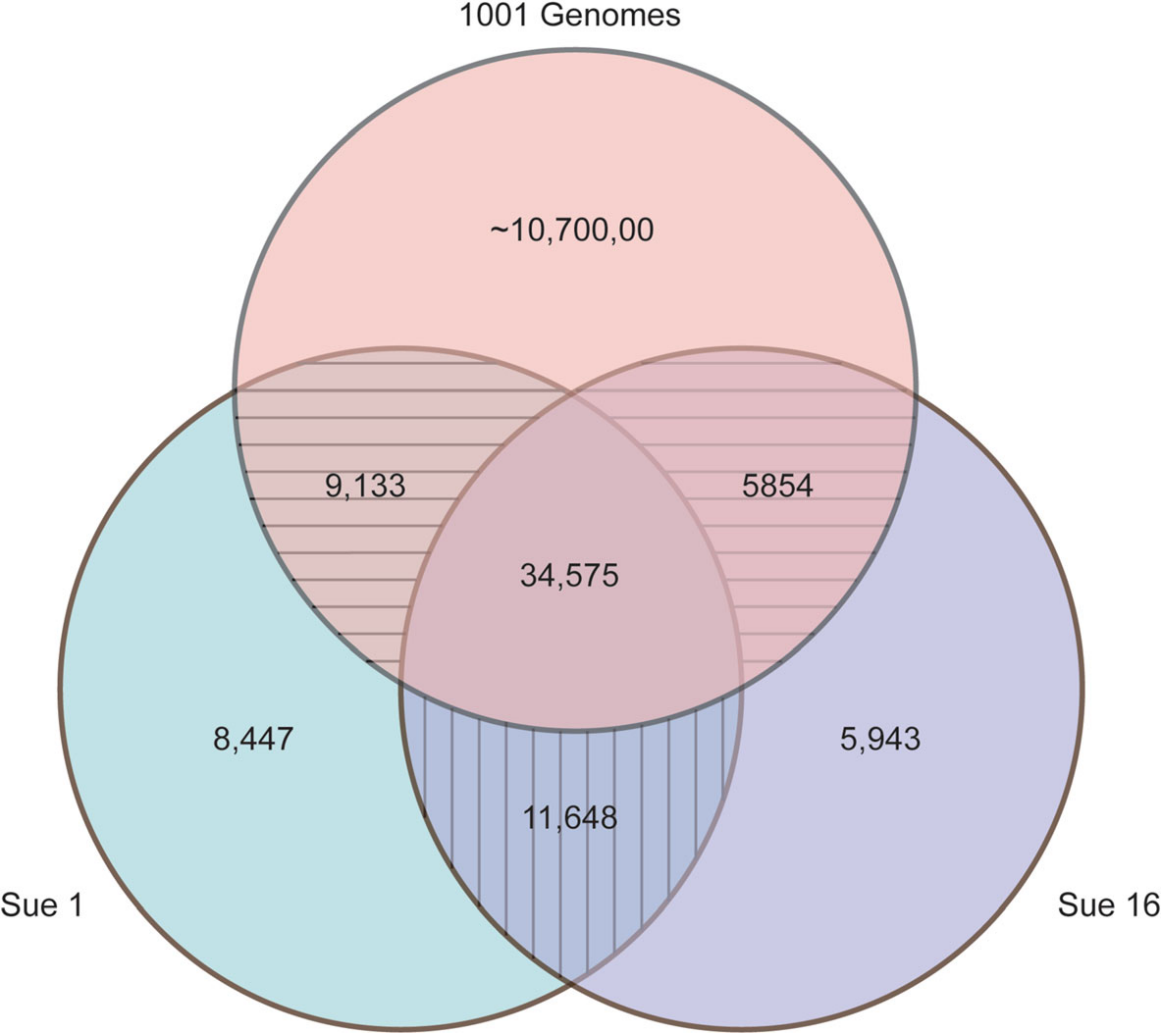
Allopolyploids Sue 1 and Sue 16 derive from the same *A. thaliana* parent, and have accumulated both private SNPs and SNPs shared between each other but not the 1001 Genome data set. SNPs for the *A. thaliana* subgenome were called against the Col reference genome

We used the transcriptome data also to call SNPs between the two accessions Sue 1 and Sue 16, and between them and their maternal *A. thaliana* parent within the gene space. Using *A. thaliana* (Col-0, TAIR10) as the reference genome we compared both the Sue 1 and Sue 16 *A. thaliana-*derived subgenomes (designated as A. sue - AT) to the *A. thaliana* reference and found 71,398 and 64,370 SNPs and indels for A. sue 1 - AT and A. sue 16 -AT, respectively. Without the indels there were 63,803 and 58,020 SNPs between the two accessions and the reference genome, respectively. When comparing A. sue 1 -AT and A. sue 16 -AT to each other, 46,223 were shared SNPs between both accessions and the Col-0 reference. A. sue 1 –At had 17,580 SNPs not found in A. sue 16 – AT, and A. sue 16 – AT had 11,797 SNPs not found in A. sue 1 – AT (Fig. 4).

We determined how many of the SNPs were private, i.e. were only found in one population sample [47]. To determine the number of private SNPs we compared SNPs for both accessions separately and together with those in the 1001 genomes (1001genomes.org) SNP dataset (Fig. 4). We found 20,095 and 17,591 SNPs in A. sue 1- AT and A. sue 16-AT, respectively, which are not found in the 1001 genomes (Fig. 4, Additional file 1: Table S5). Of those 8447 and 5943 are private SNPs found only in one of the two *A. suecica* accessions, Sue 1 and Sue 16, respectively, and not in any *A. thaliana* accession from the 1001 genomes set. These are likely SNPs that arose as Sue 1 and Sue 16 diverged from each other. Another set of SNPs contains those SNPs that are shared between each *A. suecica* accession and the 1001 genomes set but not between the two *A. suecica* accessions (horizontally hatched groups in Fig. 4). SNPs in these groups may also represent de novo SNPs that were acquired in each *A. suecica* accession as they were diverging from each other, and also independently arose in the 1001 genome set. A final set of 11,648 SNPs shared between A. sue 1 and A. sue 16 but not the 1001 genomes (vertically hatched area in Fig. 4) may have a) been present in the *A. thaliana* parent of these *A. suecica* accessions but were not represented in the 1001 genomes SNP set, b) formed immediately during (or as a result of) polyploidization, or c) evolved after polyploidization but before the divergence of Sue 1 and Sue 16. All other SNPs found in our analysis were shared with at least one known accession from the 1001 genomes data set (Fig. 4), and could therefore potentially have been inherited prior to polyploidzation.

Comparison with the 1001 genomes data set suggested that, from among the represented accessions, the ecotypes with the greatest similarity to the *A. thaliana* genome in Sue1 and Sue16 were from Russia, just north of Kazakhstan, Kyrgyzstan, and the northern-most part of Afghanistan (Additional file 1: Table S6). The ecotype with the greatest similarity for both Sue 1 and Sue 16 was the ecotype Borsk-2 (1001 Genomes ID: 9957). In the comparison of the 1001 Genomes data collection to A. sue 1-AT and A. sue 16-AT the same set of ecotypes was found among the 15 top-scoring accessions. Overall similarity of the A. sue -AT complements to these ecotypes was relatively low (Additional file 1: Table S6), as would be expected for a genome that has evolved separately from its common ancestor. This analysis supports the idea that Sue 1 and Sue 16 are sister lines from the same polyploidization event with the same *A. thaliana* parent.

## Discussion

Diversification between populations can occur via genetic changes and in the regulation of their transcriptomes. To compare differences in gene expression and estimate the genetic divergence between Sue 1 and Sue 16 we conducted RNAseq analysis. Given the species’ relatively recent formation we expected a low number of differentially expressed genes between the two accessions. We further expected that any genomic divergence between accessions would be mostly randomly distributed throughout the genome and any differentially expressed genes would not likely be enriched in particular functional categories. Contrary to our expectations, we found that the majority of differentially expressed genes showed significant enrichment in certain functional categories, specifically those related to stress (Additional file 1: Table S3). A trivial explanation would be that the plants somehow had suffered from insect damage during growth and before harvest and responded by deploying a suite of stress responses. Given the randomized arrangement of the plants in the planting trays, the use of the same soil batch for all pots, and the fact that all plants were grown simultaneously and on the same plant growth rack side by side made it unlikely that winged insects or soil contaminants could explain the observed differences. Instead, it is possible that the accessions differ from each other in stress sensitivity or that one accession displays a mild “chronic stress” state, compared to the other one, although neither one of the accessions appeared more stressed than the other when inspected visually. Because Sue 1 seems to upregulate abiotic stress response genes relative to Sue 16, while Sue 16 seems to upregulate biotic and general stress response genes (Fig. 1), it is possible that both are in a mild “chronic stress” state but are adapted to different stressors, which might suggest local adaptation of these lines to specific environments.

When further analyzing the set of differentially expressed genes we noticed that 42 of them were regulated pairwise in the same way in both subgenomes of the allopolyploid (Additional file 1: Table S2), suggesting that identical regulation of homoeologs might have provided an adaptive advantage to the population. Polyploidy is an extreme example of a gene duplication event. Gene duplications originally lead to functional redundancy, which over time is often followed by the accumulation of degenerate mutations [48]. Changes of gene function in duplicated genes can lead to subfunctionalization, where the function of the original duplicated gene is partitioned among the duplicates, or neofunctionalization, where favorable mutations in one or both copies of the duplicate take on a new function [49–51]. Interestingly, in the case of the above mentioned 42 DEGs, subfunctionalization does not occur, and on the contrary, the homoeologs are regulated synchronously. Transcriptionally stable differences in gene activity between the two allopolyploid accessions studied here might indicate the beginning of differential adaptation among a small set of the duplicated homoeologs.

We identified a set of genes that together might contribute to the phenotypic differences between Sue 1 and Sue 16, including flowering time and plant height (Fig. 3). In our scenario, increased levels of *LTP3/4* in Sue 1 lead to increased ABA biosynthesis, and thus promote stomatal closure. At the same time the increased levels of *CML23/24* in Sue 16 lead to reduced levels of NO (nitric oxide), a signaling compound that also is involved in inducing stomatal closure [43]. A lower level of NO thus reduces stomatal closure in Sue 16. Possibly acting on the same pathway, increased induction of stomatal closure in Sue 1 and reduced induction of stomatal closure in Sue 16 would have the same physiological effect. Adult Sue 1 is smaller in stature compared to Sue 16 [32], albeit not at the 4-week stage when RNA for this experiment was harvested. However, if Sue 1 has consistently elevated levels of ABA and Sue 16 has consistently lower levels of NO, this state might over time contribute to a chronic difference in transpiration efficiency and could affect carbon assimilation and growth rate, although these interactions are far from straight forward [52]. Additionally, *cml24* mutants flower late, thus it is possible that the lower expression of *CML24* (and one homoeolog of *CML23*) in Sue 1 is related to its late flowering phenotype [45]. Finally, two xyloglucan transglycosylases/hydrolases were more highly expressed in Sue 16 compared to Sue 1 (Additional file 2: Figure S1). *XTHs* play a role in cell wall modification in response to environmental signals and facilitate increased organ growth, mostly by cell elongation [46, 53]. *XTH22* specifically is responsive to canopy shading and involvement in the shade avoidance response [53, 54] - an accelerated growth response that allows plants to elongate hypocotyls or petioles to grow out of the shade or from underneath a leaf canopy. *XTH4* has been implicated in hypocotyl elongation [55]. Greater expression of these two genes in Sue 16 could thus further explain the accelerated growth in this accession. Taken together small effects from these six genes could underlie the subtle phenotypic differences observed in *A. suecica* accessions.

The results from our RNAseq analysis provide new testable hypotheses regarding the phenotypic and physiological divergence between the two lines. We have started experiments testing the hypothesis that Sue 1 and Sue 16 display differing drought responses. We have observed pronounced and statistically significant differences to prolonged drought in our initial trials, however, these experiments require more careful tests and analyses, and will be continued in the future.

It should be noted that it is of course possible that more genes than those described here are in the early stages of transcriptional divergence between accessions but were undetected in our study because gene expression differences are not obvious under the conditions tested, or because the *A. arenosa* derived copy did not map equally well to the *A. lyrata* reference as the *A. thaliana* derived copy did to its reference and thus eluded analysis.

Expression level dominance of one or the other parental genomes has been described in several allopolyploid species [56]. In our study, we noted with interest that the overall relative expression of the *A. thaliana* homoeologs was almost two-fold greater than the overall relative expression of *A. arenosa* homoeologs (Additional file 3: Figure S2). While it is possible that some of the bias is caused by the fact that our synthetic reference genome was constructed using *A. lyrata* sequences, we feel that this alone is unlikely to explain the bias. The observation is particularly interesting with respect to the fact that *A. suecica* looks much more like *A. arenosa,* repressing the *A. thaliana* phenotype in the allopolyploid. Microarray analysis of re-synthesized *A. suecica*-like allopolyploids had shown that more than 94% of genes that were down-regulated in allopolyploids were genes that were normally more strongly expressed in the *A. thaliana* parent compared to the *A. arenosa* parent, suggesting a repression of the *A. thaliana* homoeologs in the allopolyploid [10]. Although these two studies do not compare directly, it is interesting to see that in our study overall the *A. thaliana-*derived transcriptome does not appear to be repressed, and in fact may show expression level dominance in established natural *A. suecica* (Additional file 3: Figure S2).

While the exact provenance of these strains is unknown it is possible that dispersal into different microenvironments could have provided the selective conditions for regulatory changes in a small network of genes leading to different responses. We recently showed that small changes in light intensity can provide the allopolyploid *A. suecica* an advantage in photosynthetic assimilation over its parent species [57]; it is therefore easy to imagine that diverging duplicated genomes could fine tune an adaptive response to slightly different environmental conditions. Mutations in *trans*-acting transcription factors might be responsible for the synchronous regulation of both homoeologs in allopolyploids [58, 59]. Changing regulatory patterns of two homoeologous genes with one *trans*-acting genetic change might be a faster and more efficient way for evolution to proceed in allopolyploids compared to diploids. While our model built on transcriptome data (Fig. 3) is more likely to suggest new testable hypotheses than to provide a definitive answer as to the reason for the differences in phenotype, it suggests that changes in the regulation of homoeologs might be a fast lane to adaptive change.

Transcriptomic change between any two populations, and these two allopolyploid accessions in particular, can be the result of genetic, or epigenetic change, or can be due to plasticity. We used the RNA-seq data to determine how much genetic variation there was between the two lines (Fig. 4) and found a significant number of SNPs that are not present in the 1001 Genomes SNP collection, suggesting that these SNPs, which separate Sue 1 and Sue 16 might provide a genetic basis for the divergence in the accessions’ transcriptomes. However, we also found that these SNPs were unlikely to be causal for the transcriptional differences (Additional file 1: Table S6). A recent study [30] found evidence that *A. suecica* likely formed more than once in its evolutionary past. Our SNP data suggest that Sue 1 and Sue 16 are likely from the same polyploidization event. The SNPMatch analysis of the 1001 Genomes data set allowed us to propose a set of possible ecotypes, which had common ancestors with the accession that contributed its genome in the original allopolyploidization event creating the *A. suecica* ancestor line to Sue1 and Sue16 (Additional file 1: Table S3)*.* The current geographic distribution of these ecotypes, which all cluster in central Asia, is in concordance with previous and recent suggestions [30, 60] that *A. suecica* formed in Eurasia after the end of the last ice age and moved northwards with the receding ice sheet to its current distribution range.

## Conclusions

Taken together, our data suggest that genomic changes within the gene space during or after allopolyploidization coincide with changes in the transcriptome of the studied accessions. A likely explanation for the large number of coordinately differentially expressed genes from homoeologous gene pairs is that mutations in trans-acting factors can more efficiently effect gene expression in allopolyploids. Diversification in expression levels of potentially adaptive genes between accessions might contribute to the observed phenotypic variation. Many of the differentially expressed genes affect water movement in the plant, suggesting the possibility of differential niche adaptation in these two accessions. Given the large number of stress-related genes in our set of differentially expressed genes, it will also be interesting in the future to experimentally test if these genes can be used to predict differential phenotypes under specific environmental conditions.

## Additional files

Additional file 1: **Table S1.** Sequence Summary Statistics. **Table S2.** Differentially expressed genes between Sue 1 and Sue 16. Homoeologs are indicated in matching color pairs. **Table S3.** Enriched Gene Ontology Categories in 148 Differentially Expressed Genes. Analysis was performed using topGO in R (Alexa and Rahnenfuhrer [36]). *P*-values are from Fisher’s Exact Test under a weighted model (Alexa et al. [37]). We further categorized the GO categories into either biotic stress response, abiotic stress response, general stress response (both biotic and abiotic), or not stress related categories. **Table S4.** Observed SNP distribution in DEGs and non-differentially expressed genes. Total basepairs (bps) in coding sequence (CDS) in the bottom right cell came from the *A. thaliana* CDS FASTA file. Of those, 155,619 bps fall in differentially expressed genes (DEGs). Our analysis found 71,636 SNPs between Sue 1 and Sue 16,203 of which fall in DEGs. We performed a chi-square analysis and got a *p*-value of .32 indicating there is no difference from the expected distribution if SNPs fell randomly in all genes. **Table S5.** SNPs in Sue 1, Sue 16, and the 1001 genomes database. **Table S6.** Comparison of Sue 1 and Sue 16 SNPs to ecotypes in the 1001 genomes database using SNPmatch. (XLSX 36 kb)

Additional file 2: **Figure S1.** Members of the xyloglucan transglycosylase/hydrolase (XTH) family are significantly differentially upregulated in Sue 16 relative to Sue 1. Shown are FPKM expression values with confidence intervals from Cuffdiff analysis for both homoeologs of *XTH4* and *XTH22*. (PDF 471 kb)

Additional file 3: **Figure S2.** In *A. suecica* the homoeologs of the *A. arenosa* subgenome are more highly expressed than the homoeologs of the *A. thaliana* subgenome. Relative homoeolog expression of AT homoeolog to AA homoelog in Sue 1 and Sue 16 was calculated. For the 13,394 homoeologous genes analyzed (with the exception of those with FPKMs of zero) the log2 ratio of the average AT homoeolog FPKM to the average AA homoeolog FPKM is plotted in a density graph. While overall expression levels between Sue 1 and Sue 16 are similar, the analysis suggests that the AA subgenome is overall about twice as much expressed as the AT subgenome. The line at zero indicates where homoeologs have equivalent expression. (PDF 548 kb)

Additional file 4: **Table S7.** Complete RNA seq data. (TXT 9489 kb)

## Abbreviations

AA: *Arabidopsis arenosa*
ABA: Abscisic acid
AT: *Arabidopsis thaliana*
DEG: Differentially expressed genes
FPKM: Fragments Per Kilobase of transcript per Million mapped reads
GO: Gene ontology
NO: Nitric oxide
SNP: Single nucleotide polymorphism
TAIR: The arabidopsis information resource
