# Supplementary figures and images for: Natural variation in stress response gene activity in the allopolyploid Arabidopsis suecica
Source: BMC Genomics. 2017 Aug 23;18:653. doi: 10.1186/s12864-017-4067-x (PMC5567635; doi:10.1186/s12864-017-4067-x)

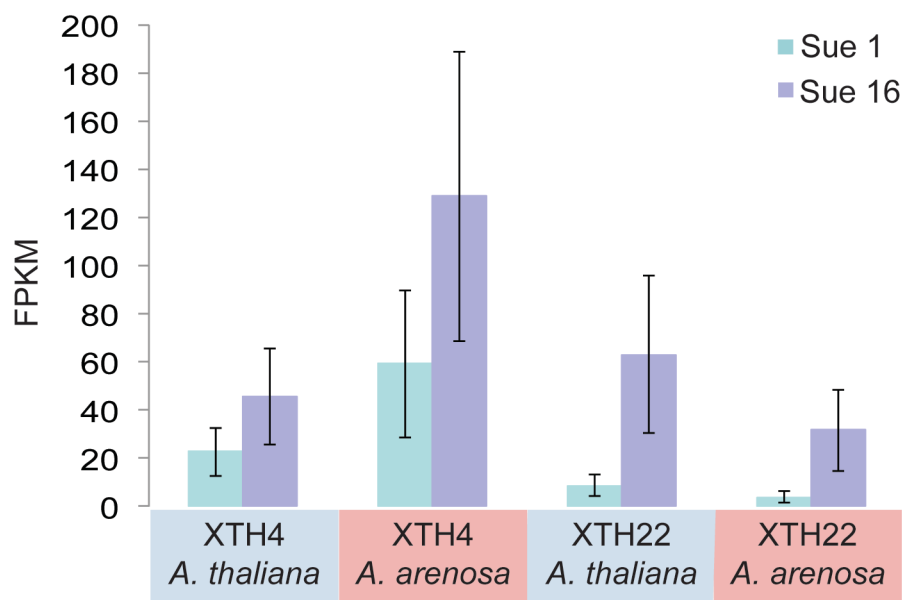

Supplement: Supplementary file 2 — Figure S1. Members of the xyloglucan transglycosylase/hydrolase (XTH) family are significantly differentially upregulated in Sue 16 relative to Sue 1. Shown are FPKM expression values with confidence intervals from Cuffdiff analysis for both homoeologs of XTH4 and XTH22. (PDF 471 kb) [file 12864_2017_4067_MOESM2_ESM.pdf]

Relative Homoeolog Expression in *A. suecica*

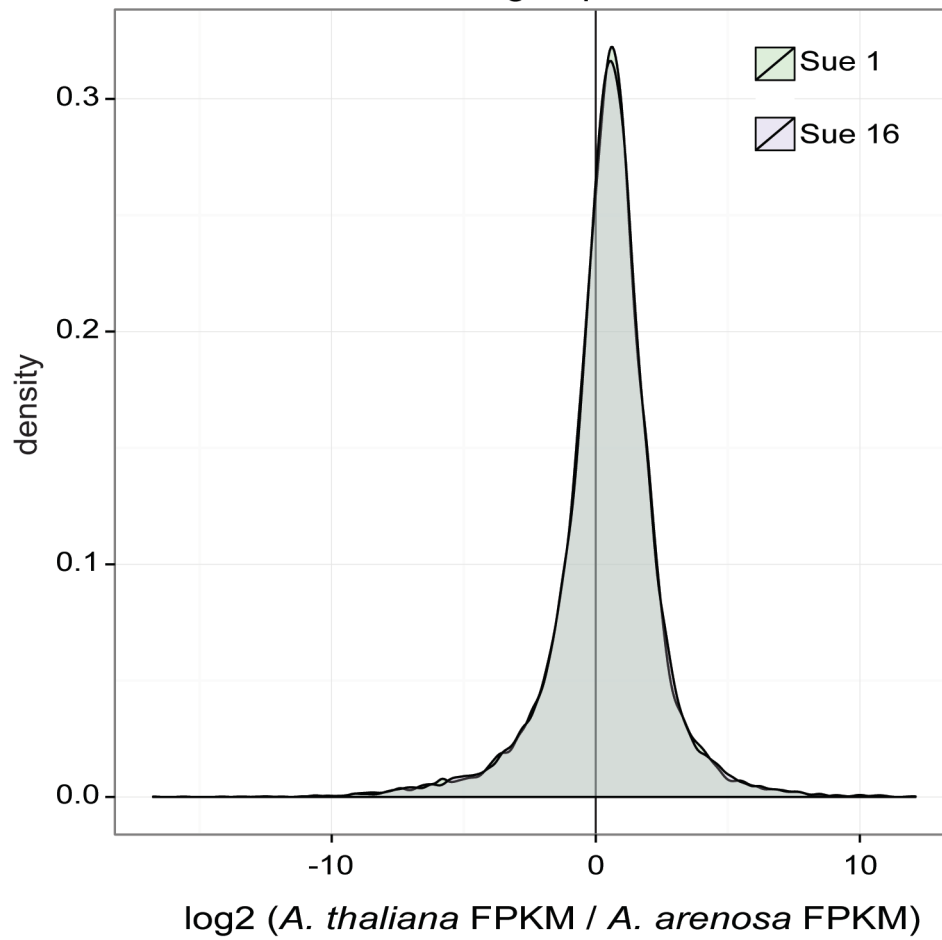

Supplement: Supplementary file 3 — Figure S2. In A. suecica the homoeologs of the A. arenosa subgenome are more highly expressed than the homoeologs of the A. thaliana subgenome. Relative homoeolog expression of AT homoeolog to AA homoelog in Sue 1 and Sue 16 was calculated. For the 13,394 homoeologous genes analyzed (with the exception of those with FPKMs of zero) the log2 ratio of the average AT homoeolog FPKM to the average AA homoeolog FPKM is plotted in a density graph. While overall expression levels between Sue 1 and Sue 16 are similar, the analysis suggests that the AA subgenome is overall about twice as much expressed as the AT subgenome. The line at zero indicates where homoeologs have equivalent expression. (PDF 548 kb) [file 12864_2017_4067_MOESM3_ESM.pdf]
